# Tracheal Diverticulum Mimicking Pneumomediastinum: A Case Report Emphasizing the Importance of Differential Diagnosis in Chest Imaging Evaluation

**DOI:** 10.7759/cureus.39297

**Published:** 2023-05-21

**Authors:** Manveer K Ubhi, Luis E Irizarry Nieves, Rainier Michael R Cabatbat, Teresa Del Rio, Mukosolu F Obi, Parvez Mir

**Affiliations:** 1 Internal Medicine, Wyckoff Heights Medical Center, Brooklyn, USA; 2 Internal Medicine and Pulmonology, Wyckoff Heights Medical Center, Brooklyn, USA

**Keywords:** pneumomediastinum, idiopathic hemidiaphragm paralysis, radio-graphic finding, congenital abnormality, pulmonary disease, tracheal diverticulum

## Abstract

A tracheal diverticulum (TD) is a generally benign medical condition, where there is an outpouching of the tracheal wall. Additionally, it is generally asymptomatic but there have been reported cases of adverse outcomes linked to TD. Here we present a case of a tracheal diverticulum that was incidentally found during the workup for stroke in a patient. Moreover, due to its radiologic appearance, there was concern for pneumomediastinum. We highlight the presentation and clinical importance of TD owing to the complications one is predisposed to from having this condition.

## Introduction

A tracheal diverticulum (TD) is considered an uncommonly diagnosed medical condition in which there can be an outpouching of the tracheal wall with a currently reported prevalence of 2.4-8.1% [1]. TD may be separated into two categories: congenital and acquired. Congenital TD is generally made of all layers of the tracheal wall (respiratory epithelium, smooth muscle, and cartilage) and located below the vocal cords to just above the carina, whereas an acquired TD has no involvement of the smooth muscle or cartilage. Comparatively, congenital TD is larger in size and has larger communication with the trachea. While congenital diverticulum is due to developmental failure, the development of an acquired TD is associated with chronic increases in intraluminal pressure, such as in chronic obstructive pulmonary disease (COPD), which leads to the weakening of the tracheal wall [1]. Although TDs are considered benign in nature, they are occasionally symptomatic, presenting with cough, dyspnea, frequent respiratory infections, dysphonia, or hiccups [2-4]. Additionally, there have been several reported cases of adverse outcomes associated with TD associated with ventilatory support [5-7]. This is especially concerning in patients with acquired TD as they are generally a population with chronic lung problems that lead to sometimes invasive ventilatory intervention [1]. 

We present a case of an incidental finding of TD during a stroke/transient ischemic attack (TIA) evaluation in the emergency department in hopes of highlighting awareness of TD given that chest imaging is now ubiquitous in clinical practice and the importance of documentation of this condition for managing patients given reported associated adverse complications.

## Case presentation

**Figure 1:**
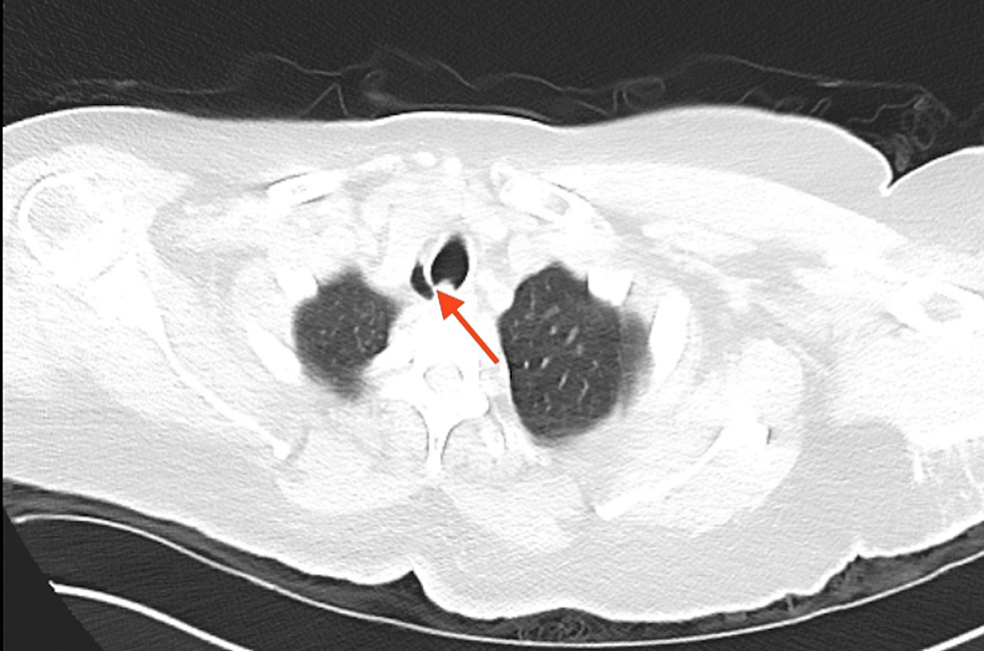
Computed tomography (CT) of the chest without contrast, axial lung window. Red arrow points to tracheal diverticulum, lateral to trachea.

**Figure 2:**
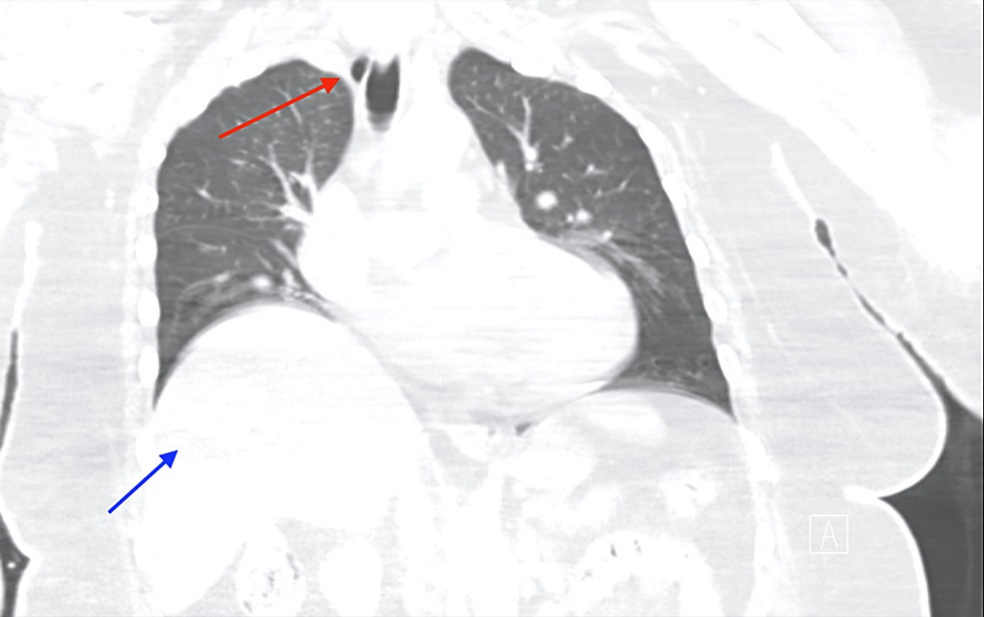
Computed tomography (CT) of the chest without contrast, coronal lung window. Red arrow points to tracheal diverticulum, lateral to trachea, and blue arrow points to right hemidiaphragm.

This is a case of a 59-year-old female with a known past medical history of asthma, schizophrenia, and Parkinson's disease, who was brought into the hospital with the chief concern of lightheadedness. She presented with associated nausea, left-sided headache, and worsening body stiffness with an inability to ambulate for five days. Upon presenting to the emergency department, the patient was worked up for a stroke. Computed tomography (CT) of the head, CT angio of the head and neck, and CT perfusion were performed, which ruled out an acute cerebrovascular accident; however, on the CT angiogram of the head and neck, she was found to have an incidental finding of pneumomediastinum in the right superior thoracic cavity without extension into the neck. She denied having a history of shortness of breath, coughing spells, nausea, or vomiting associated with retching and denied any substance use. Pulmonary services were consulted and a dedicated CT chest was performed, which suggested the air pocket was likely a congenital TD (Figures 1, 2) and right hemidiaphragm (Figure 2). No previous images were available for comparison. No intervention was required as she was asymptomatic. The TD finding was explained to her, and the finding was documented in her medical record for future reference.

## Discussion

TD, a type of paratracheal air cyst, is characterized as a benign out-pouching of the tracheal wall lined by ciliated columnar epithelium [1,8]. Typically an auxiliary finding on imaging studies with an approximate incidence of 2.4%, TD rarely warrants intervention [9]. TD forms one subgroup of the many anatomical thoracic variants, which includes cardiac bronchus, thoracic bronchus, and azygous lobe with a prevalence of 0%, 0.2%, and 0.8%, respectively [10]. The current diagnostic approach to TD involves a thorough clinical evaluation, imaging studies, and, in some cases, bronchoscopy. Bronchoscopy may be necessary in cases where the diagnosis is unclear, or to assess the extent of the TD. A recent retrospective study on the use of magnetic resonance imaging (MRI) in the evaluation of TD showed that it was useful in providing additional information about the wall, unable to be seen on CT. It also showed that the MRI was superior in evaluating for infection and response to therapy in cases of infected TD [11]. TD is most frequently located posterolaterally, on the right, at the level of Th1-Th3, likely because this area is unprotected while this corresponding area on the left is protected by both the aortic arch and esophagus [12]. While tracheal TDs are often incidental and asymptomatic, it is not uncommon that they can be misdiagnosed as pneumomediastinum [13]. TD has also been reported in a case of spontaneous pneumomediastinum (SPM) in a 25-year-old asthmatic patient who was later discovered to have a TD [14]. Despite not having a history of penetrating trauma, this patient had a history of asthma which, given its association with SPM, validates the suspicion for a pneumomediastinum.

Although rare, TD can be symptomatic, manifesting as recurrent respiratory tract infections since the cavitation becomes a nidus for infections. Chronic cough, hemoptysis, dyspnea, stridor, and dysphonia are among the other symptoms that are associated with TD [15]. The presence or absence of symptoms dictates the management of TD. Small, asymptomatic TD may not require any treatment and can be managed with observation, as in our current patient. Surgical resection is the preferred treatment modality for symptomatic cases. Endoscopic resection or thoracoscopic resection may be used to remove the TD. In the case of our patient, no management was required, but it is important to include this finding in her history, given the reported complications associated with TD. 

Current literature also revealed that TD predisposes patients to significant complications and/or may be associated with worse outcomes. Potential complications include infections that may even progress to paratracheal abscesses or empyema secondary to recurrent upper respiratory tract infections [1,16]. The presence of TD can also complicate interventions such as tracheal intubation and administration of positive pressure ventilation since the TD is prone to perforation, giving rise to pneumomediastinum [17]. A literature review has also revealed cases reported of TD arising after non-invasive ventilation in coronavirus disease 2019 (COVID-19) patients, which had an associated pneumomediastinum [5]. Regarding this case, no complications or symptoms were reported after the incidental finding was determined to not be a pneumomediastinum but instead a TD. We highlight the importance of this differential diagnosis in the evaluation of imaging because, despite the benign course, TD may have implications for the clinical course of the patient.

## Conclusions

Often incidental and asymptomatic, TD can be misdiagnosed as pneumomediastinum in imaging studies. In the rare instance a TD is symptomatic, it may present as recurrent respiratory tract infections or chronic cough, therefore warranting further evaluation and treatment. If the patient is asymptomatic, treatment is not indicated. However, despite its benign course, it is recommended to include TD as a differential diagnosis in the evaluation of imaging given its complications.
